# An improved method to efficiently acquire rice black-streaked dwarf virus viruliferous small brown planthoppers

**DOI:** 10.3389/fgene.2023.1111030

**Published:** 2023-02-01

**Authors:** Linlin Du, Bo Zeng, Xuejuan Li, Ying Lan, Wei Guo, Zhaoyun Wang, Zhiyang Liu, Yijun Zhou, Kumar Kunda Jiban, Tong Zhou

**Affiliations:** ^1^ College of Plant Protection, Nanjing Agricultural University, Nanjing, China; ^2^ Jiangsu Key Laboratory for Food Quality and Safety-State Key Laboratory Cultivation Base of Ministry of Science and Technology/International Rice Research Institute and Jiangsu Academy of Agricultural Sciences Joint Laboratory/Institute of Plant Protection, Jiangsu Academy of Agricultural Sciences, Nanjing, China; ^3^ National Agricultural Technology Extension Service Center, Beijing, China; ^4^ Plant Virus and Vector Interactions, Centre for Plant Virus Research, Crop Research Institute, Prague, Czechia

**Keywords:** RBSDV, viruliferous SBPHs, feeding times, RT-qPCR, virus loads

## Abstract

Accurate phenotypic identification is the basis of research for resistance genetics and rice breeding for resistance to RBSDV disease. Obtaining rice black-streaked dwarf virus (RBSDV) viruliferous small brown planthoppers (SBPHs) with high transmission efficiency is an essential part of accurate phenotypic identification. Here, through quantifying number of RBSDV copies in infected rice plants, optimizing times of SBPHs fed on RBSDV-infected rice plants and leaf stage of rice seedlings, a method to acquire an RBSDV-carrying SBPH population more efficiently was improved. The results showed that rate of viruliferous SBPHs was significantly higher when fed on RBSDV-infected rice plants that had the copy numbers of RBSDV S10 of 3.0*10^4^ and 1.1*10^4^ than 8.3*10^2^. Therefore, it is more efficient for SBPHs to acquire the virus when fed on RBSDV-infected rice plants that have copy numbers of RBSDV S10 above 1.1*10^4^. The rate of viruliferous SBPHs were 50% and 54%, respectively, after the insects fed on RBSDV-infected rice plants for 7 and 9 days and being transferred to healthy rice seedlings for 5 and 3 days, which was significantly higher than those at other feeding times. The optimal inoculation leaf stage of rice seedlings was the 2-3-leaf stage (3 effective SBPHs per seedling for 72 h), but a high rate of viruliferous SBPHs may be suggested for inoculation of older rice seedlings.

## Introduction

Rice black-streaked dwarf virus (RBSDV) belongs to the genus *Fijivirus*, family Spinareoviridae (Wu et al., 2020; Matthijnssens et al., 2022) of the order *Reovirales*. RBSDV is transmitted by small brown planthoppers (SBPHs; *Laodelphax striatellus*) in a persistent and propagative manner but not through their eggs (Ruan et al., 1984). SBPHs can also transmit rice stripe virus (RSV) to rice and can be transmitted to SBPH offspring through transovarian transmission (Falk and Tsai, 1998). RBSDV can cause severe damage to rice and maize production, and RBSDV has been shown to also infect wheat, barley, and other cereal crop species in East Asia (Zheng et al., 2012; Zhou et al., 2017). Typical symptoms caused by RBSDV on rice plants include extreme stunning, dark-green leaves, twisting of distal parts of leaves and fewer tillers. At the later stage of the disease, waxy enations appear on the veins, the outer sides of leaf sheaths and on the stem (Tao et al., 2013).

As there are no known infectious clones of RBSDV, the method of inoculation on rice plants relies on the virus vectors SBPHs. RBSDV cannot be transmitted to the progeny of SBPHs through transovarian transmission. Therefore, RBSDV viruliferous SBPHs are commonly obtained by allowing virus-free nymphs to feed on RBSDV-infected rice plants. RBSDV-transmissible SBPHs are important for laying a foundation for breeding resistant varieties, uncovering the mechanism of RBSDV pathogenesis on rice plants and effectively preventing and controlling the disease. In previous studies, most of the RBSDV inoculation experiments were conducted under natural conditions. The rate of viruliferous SBPHs in these experiments was not the same because the same conditions could not be maintained in the natural fields (the population of SBPHs, the growth state of SBPHs, the state of rice plant growth) (Wang et al., 2010), which could lead to unrepeatable results (Li et al., 2008; Pan et al., 2009; Wang et al., 2010). A method of artificial inoculation identification of rice cultivar resistance to rice black-streaked dwarf disease was established (Zhou et al., 2011a). SBPH nymphs were allowed to feed on RBSDV-infected rice plants for 2–3 days and then transferred onto healthy rice seedlings of cultivar Wuyujing No. 3 for another 10–12 days to acquire RBSDV. By the use of this method, the rate of viruliferous SBPHs was usually determined to be 20%–35%. Each rice plant was inoculated with two to six virus-carrying SBPHs for 2–3 days, thus causing RBSDV disease in the rice plants (Zhou et al., 2011b). Using this artificial inoculation method, we found that the viruliferous rate was also variable (Sun et al., 2013; Li et al., 2017).

In particular, a low viruliferous rate of the insects would result in a large amount of SBPHs, which means that a larger population would be prepared and that fewer rice plants would be inoculated in the identification experiment. Therefore, the increase in the viruliferous population for SBPHs is essential. In this study, we improved a method to acquire an RBSDV-carrying SBPH population more efficiently.

## Results

### Virus concentration in RBSDV-infected rice plants

**FIGURE 1:**
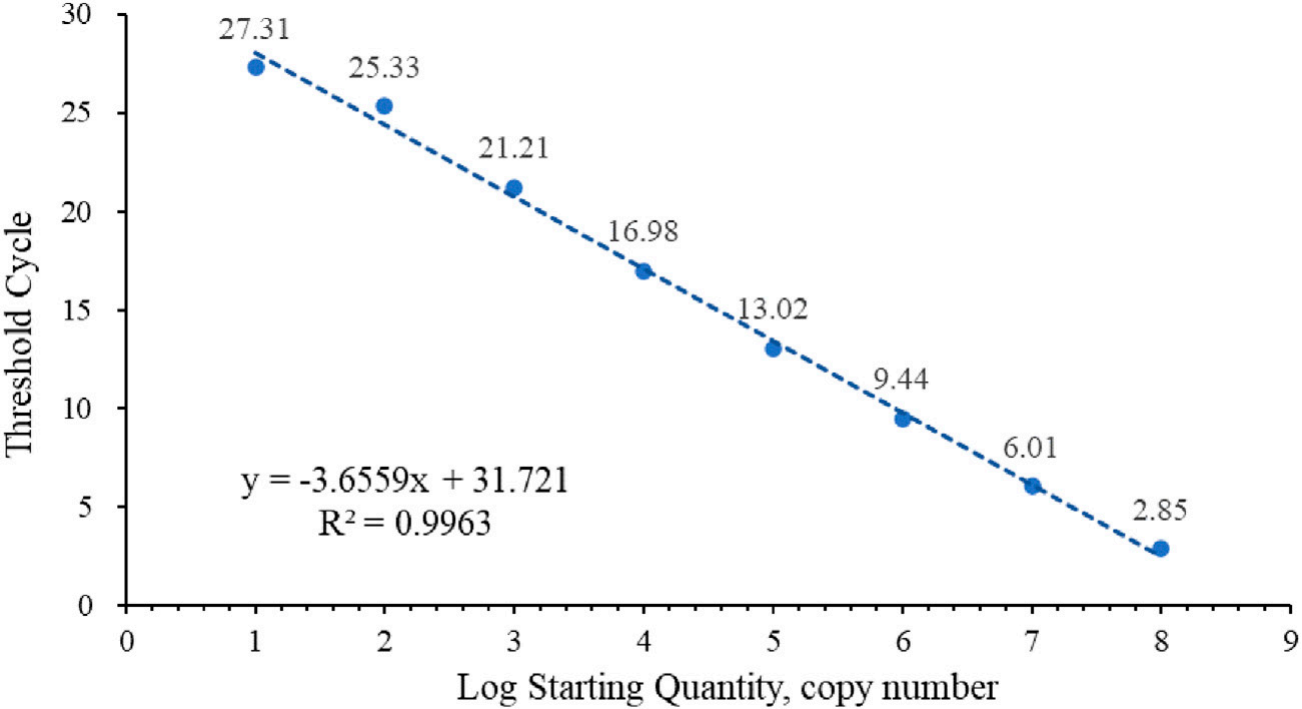
Standard curve of RBSDV-S10.

The standard curve for quantifying the absolute number of RBSDV copies in infected rice was determined using 10-fold serial dilutions of RNA standards ranging from 1.0 × 10^9^ to 1.0 × 10^2^ copies/reaction (Figure 1). Cycle threshold (Ct) values were measured and plotted against the known copy numbers of the standard sample. The standard curve covered a linear range of eight orders of magnitude. The slope (−3.6559) and correlation coefficient (*R* ^2^ = 0.9963) of the standard curve indicated that this assay can be used to quantify target RNA in infected rice tissue.

**FIGURE 2:**
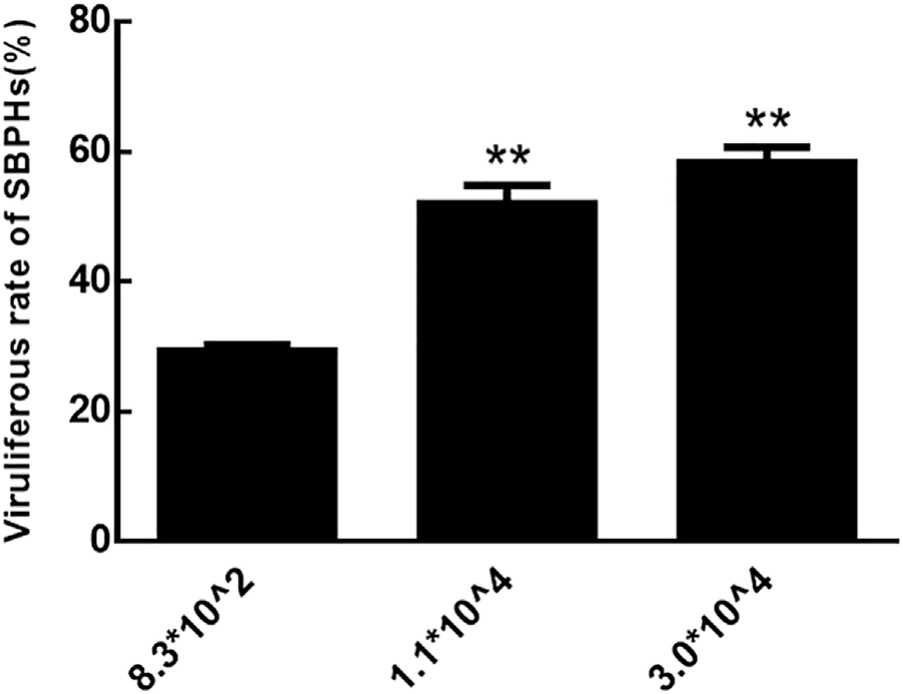
Viruliferous rate of SBPHs that fed on RBSDV-infected rice plants with different RBSDV contents.

The rate of viruliferous SBPHs that fed on RBSDV-infected rice plants with copy numbers of RBSDV S10 3.0*10^4^ and 1.1*10^4^ was significantly higher than those that fed on plants with numbers of 8.3*10^2^ (Figure 2). Therefore, RBSDV-infected rice plants with a copy number of RBSDV S10 above 1.1*10^4^ were more suitable for SBPHs to obtain RBSDV.

### Optimal time of SBPHs fed on RBSDV-infected rice plants

**FIGURE 3:**
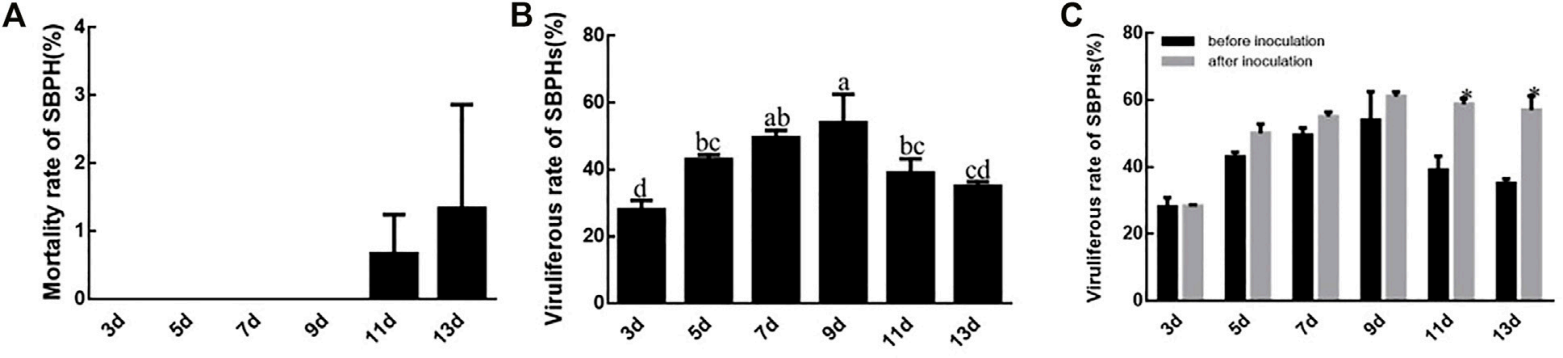
SBPH mortality and RBSDV viruliferous rates of SBPHs. **(A)**, SBPH mortality with different virus feeding times. **(B)**, RBSDV viruliferous rates of SBPHs with different virus feeding times. **(C)**, Comparison of RBSDV viruliferous rates with different virus feeding times before and after inoculation. The different letters above the columns represent statistical significance between treatments according to Duncan’s multiple tests; *p* < 0.05.

SBPHs survive without decline until the 11th or 13th day when feeding on RBSDV-infected rice plants. The mortality rate of SBPH after 13 days was significantly different from the other time points (Figure 3A). The rate of viruliferous SBPHs increased from 3 to 9 days and then decreased again, but it was still higher than after 3 days. The 9 days corresponded to the highest viruliferous rate of SBPHs (54%), which was significantly different compared to those at other feeding times except 7 days (50%). However, the viruliferous rate at 7 days was not significantly different from that at 5 days and 11 days (Figure 3B). The viruliferous rates at 3–9 days after the inoculation period did not differ from the pre-inoculation test, while the viruliferous rate at 11th day and 13th day increased after inoculation (Figure 3C).

**FIGURE 4:**
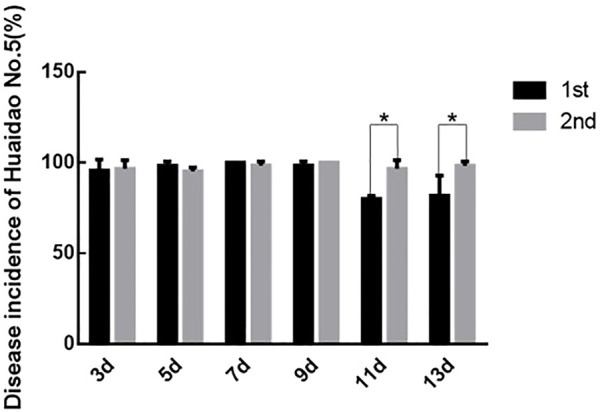
Comparison of DIs between the two inoculations. The asterisks above the columns represent statistical significance between treatments according to Duncan’s multiple tests; *p* < 0.05.

Prolongation of virus-feeding time made a difference in virus transmission and disease incidences (DIs) of Huaidao No. 5 rice. There was no difference in the DIs of rice inoculated with SBPHs feeding on the RBSDV-infected rice plants for 7 and 9 days. On the other hand, the rice DIs differed between the two rice groups inoculated with SBPHs feeding on the RBSDV-carrying rice plants for 11 and 13 days (Figure 4). Based on the above results, the optimal time for SBPHs to feed on RBSDV-infected rice plants was 7–9 days.

### Optimal inoculation leaf stage of rice seedlings

**FIGURE 5:**
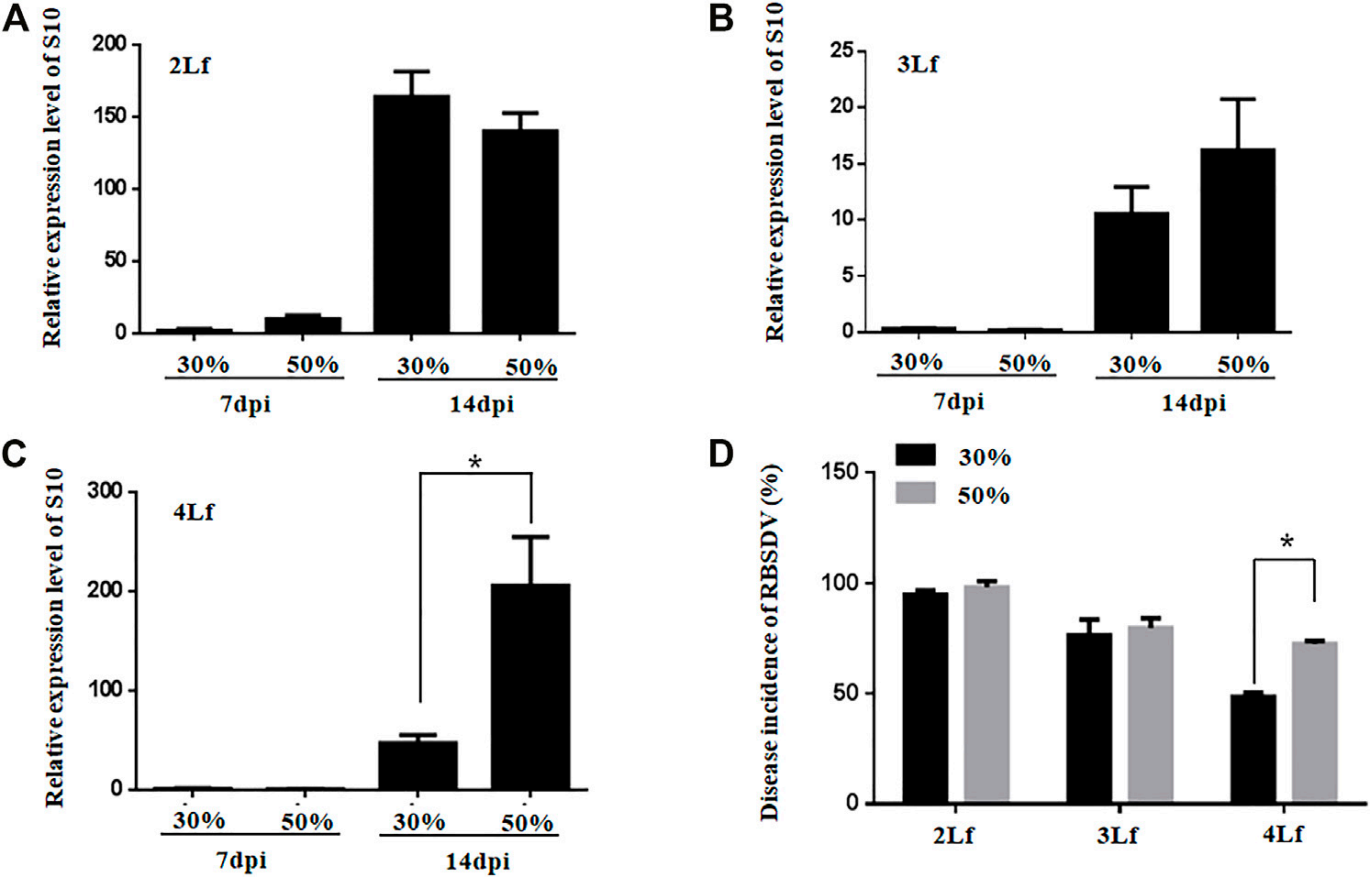
Relative expression of RBSDV S10 and the DIs of rice plants inoculated with SBPHs with different viruliferous rates at different leaf stages (Lf). **(A–C)**, Accumulation of RBSDV S10 in rice plants (2-, 3- or 4-leaf stage) inoculated with RBSDV viruliferous SBPHs with 30% or 50% viruliferous rates. **(D)**, DIs of the rice plants (2-, 3- or 4-leaf stage) inoculated with RBSDV viruliferous SBPHs with 30% or 50% viruliferous rates.

The virus loads were not significantly different between rice plants inoculated with SBPHs with RBSDV viruliferous rate of 30% and 50% at the 2-3-leaf stage (Figures 5A, B). However, the virus loads were higher in rice plants inoculated with SBPHs with 50% viruliferous rates than in those inoculated with 30% viruliferous rates at the 4-leaf stage (Figure 5C). The DIs of rice plants was higher when they were inoculated with SBPHs with a 50% viruliferous rate than with a 30% viruliferous rate at the 4-leaf stage (Figure 5D). The results suggest that the optimal inoculation leaf stage for inoculation of rice seedlings is the 2-3-leaf stage, and that a high rate of viruliferous SBPHs should be required for successful inoculation of RBSDV in the older rice plants with high efficiency.

## Discussion

In the study of plant viruses and the breeding of plant resistance in general and of insect-transmitted viruses in particular, the optimal methods of artificial inoculation are of great importance. Artificial inoculation with SBPHs is widely used for breeding rice resistance to RBSDV. Viruliferous SBPHs are one of the most important components of artificial inoculation. RBSDV is permanently transmitted by SBPH but not through their eggs (Maramorosch, 1955). Unlike rice stripe virus (RSV), it is not possible to acquire viruliferous SBPHs through continuous indoor feeding them indoors (Ren et al., 2014). Therefore, viruliferous SBPHs are acquired by allowing them to feed rice plants with classic symptoms of RBSDV disease under the conventional method. Maize is not a suitable host for SBPHs because SBPHs can hardly obtain the virus by sucking RBSDV-infected maize (Maramorosch, 1955; Qiao et al., 2009; Cao et al., 2017). Wheat and rice can be used as virus-feeding sources, and wheat seedlings can be used as intermediate hosts to maintain RBSDV in winter (Ren et al., 2014), rice plants infected with RBSDV are suitable RBSDV sources in summer and autumn.

The higher the proportion of viruliferous SBPHs was, the lower the total amount of SBPHs used, while the inoculation intensity remains the same. However, it is difficult to obtain large numbers of viruliferous SBPHs quickly because of the long feeding period and tedious and delicate operation steps. This study clearly involved selection of second-instar nymphs to start feeding on the virus-infected plants, which decreased the death of nymphs during the transmission process. The optimum combination was also defined in the study. The nymphs were allowed to feed on rice plants infected with RBSDV for 7–9 days and then transferred onto healthy rice cultivar Wuyujing No. 3 seedlings for 5 to 3 days. The rate of viruliferous SBPHs observed in this study were higher than 50%, compared to about 20%–30% in previous artificial inoculation and identification methods (Zhou et al., 2010; Zhou et al., 2011a).

The high viruliferous proportion of SBPHs leads to a low total amount of SBPHs in a rice variety, which means that more rice varieties can be identified as resistant to black-streaked dwarf virus disease at once. The rate of viruliferous SBPHs fed to RBSDV-infected rice plants differed between “before inoculation” and “after inoculation” at 11 and 13 days, which suggested that the viruliferous rate increased during the inoculation process at 11 and 13 days. There were no differences in the transmission efficiency of RBSDV between two replicates of inoculation at the feeding period of 3, 5, 7 and 9 days, but the period of 11 and 13 days. It was speculated that the prolongation of virus-feeding time influenced the mortality of SBPHs, resulting in low transmission efficiency of the virus and the appearance of the disease in the rice plants, but when the same SBPHs were used a second time for inoculation, the ability of SBPHs to transmit the virus was restored. Overall, SBPHs fed RBSDV-infected rice plants for 7–9 days were able to achieve a higher viruliferous rate and the virus concentration was relatively stable.

When second-instar SBPH nymphs were allowed to feed on RBSDV-infected rice plants with a copy number of RBSDV S10 above 1.1*10^4^ for 7–9 days and then transferred to healthy rice seedlings for 5 to 3 days, a large number of viruliferous SBPH populations were obtained. The viruliferous SBPH populations serve as materials for the identification of rice and maize resistance to RBSDV disease and maize rough dwarf disease (Zhang et al., 2001). Furthermore, the viruliferous SBPH populations can be used in plant antivirus genetic research and for elucidating molecular mechanism underlying RBSDV resistance, which promises to accelerate the development of rice breeding for resistance to RBSDV disease.

## Methods

### Sources of the virus, plant materials and vectors

RBSDV-infected rice plants were planted in the field of Jiangsu Academy of Agricultural Science. Usually, rice plants at the 6-8-leaf stage were chosen as the sources of the virus. The rice cultivar Wuyujing No. 3 was used to feed the SBPHs, and the rice cultivar Huaidao No. 5, which is susceptible to RBSDV, was used to confirm the leaf stage of rice inoculation.

The SBPHs were allowed to feed on the rice cultivar Wuyujing No. 3 across generations in a growth chamber at 25°C with a 16 h day/8 h night photoperiod (Cao et al., 2017).

### Virus concentration in rice plants infected with RBSDV

Total RNA from rice tissue was extracted using TRIzol^®^ reagent according to the manufacturer’s instructions. In the final step, the RNA was resuspended in 30 μL of diethyl phosphorocyanidate (DEPC)-treated water. The concentration of RNA was determined by spectrophotometric analysis (NanoDrop 2000C) and diluted to 1.0 μg/μL. The integrity of the RNA was subsequently assessed by 1.0% agarose gel electrophoresis. The RNA was reverse transcribed into cDNA following the instructions of the PrimeScript™ RT reagent Kit with gDNA Eraser. The primers were designed based on the highly conserved regions of RBSDV S10 (GenBank Accession no. AY050488.1) using Oligo software (Table 1).

**TABLE 1:** Primer sequences used.

Primer sequence	5'→3′
*S10*-qF	GGT​GGT​TAC​GAT​TTC​AAT​TGT​CCT​G
*S10*-qR	GCG​CTC​CAA​GTC​TGT​TCG​AGT​AAA​G

To construct the standard curve for quantifying the absolute number of RBSDV S10 copies in rice tissue, 10-fold serial dilutions of DNA standards were prepared. PCR was supplemented with 2 μL of cDNA from the abovementioned RT step and corresponding primers (0.2 μM each) in a 20 μL reaction volume. After agarose gel electrophoresis (1.5% agarose, TAE), the PCR product was purified *via* a gel extraction kit and sequenced to verify that it represented the target DNA fragment. The concentration of DNA standards (A ng/μL) was determined by spectrophotometry (NanoDrop 2000C) and converted to molecular copy number using the following formula (6.02 × 10^23^) × (A ng/μL × 10^−9^)/(DNA length×660). Further 10-fold serial dilutions of the DNA standards were performed to generate the standard curve.

Real-time PCR was performed using TB Green^®^ Premix Ex Taq™ (Tli RNaseH Plus) (Takara). Amplification was performed using the Bio-Rad IQTM^5^ Multicolor Real-Time PCR Detection System. Data were analyzed using IQ 5 Optical System software version 2.0. The absolute number of RBSDV S10 copies of rice infected with RBSDV in different time periods was determined *via* real-time PCR.

RBSDV-infected rice plants from different periods were transplanted into a 5-L glass beaker. The virus-free nymphs of the second-instar were fed to rice plants at different periods for 2–3 days to acquire the virus and then were transferred onto healthy rice seedlings of cultivar Wuyujing No. 3 (1.5–2.0 leaf stage); the insects were reared f at 25°C or 12 days to pass through the circulative period (Zhou et al., 2015). The rate of viruliferous SBPHs was subsequently detected using the Dot enzyme-linked immunosorbent assay (dot-ELISA) (Wu et al., 2013).

### Optimal feeding time of SBPHs on RBSDV-infected rice plants

Rice plants with the same RBSDV load were transplanted into a 1-L glass beaker. One hundred virus-free second-instar SBPHs nymphs were fed to these plants for 3, 5, 7, 9, 11 and 13 days. The number of dead insects was counted at each time point to determine the mortality rate of SBPHs, and the experiment was repeated three times.

Rice plants infected with RBSDV were transplanted into a 1-L glass beaker. Two hundred RSV-free second-instar SBPHs nymphs were fed with the rice plants for 3, 5, 7, 9, 11 and 13 days to obtain the virus and then transferred to healthy seedlings of rice cultivar Wuyujing No. 3 (1.5–2.0-leaf stage) for 9, 7, 5, 3, 1, and 0 days to allow the virus to pass through its circulation phase. Fifty SBPHs before inoculation at each time point were randomly selected to be assayed using dot-ELISA (Wu et al., 2013); the experiment was repeated three times. To check whether the viruliferous rate remained the same during the inoculation period, the rate of the viruliferous SBPHs was also determined after inoculation using dot-ELISA (Wu et al., 2013).

To investigate whether the virus-feeding time affects the rice DIs, after being soaked in water for 48 h and allowed to germinate for 24 h, thirty-five seeds of rice cultivar Huaidao No. 5 were sown into 1-L beakers in a growth chamber with 35%–45% humidity and a temperature between 25°C and 30°C (Cao et al., 2017). At about the 2-leaf stage, thirty healthy rice seedlings were inoculated with 3 SBPHs per seedling for 72 h (the number of SBPHs for inoculation = effective inoculation number/viruliferous% × numbers of seedlings) that were allowed to feed on RBSDV-infected rice plants for 3, 5, 7, 9, 11, and 13 days (1st). The same SBPHs were removed from another group of Huaidao No. 5 rice plants for another 3 days (2nd).

### Optimum inoculation leaf stage

Approximately thirty-five seeds of rice cultivar Huaidao No. 5 were sown as described above. At the 2, 3, and 4-leaf stages, thirty rice seedlings were inoculated individually with RBSDV viruliferous SBPHs (three effective SBPHs per seedling) for 72 h, that had been detected RBSDV viruliferous rate of 30% and 50%, respectively. The insects were gently disturbed with a brush every 12 h to force them to distperse evenly among the rice seedlings. The rice seedlings were transplanted to an experimental field with cement tank at the Jiangsu Academy of Agricultural Sciences, which were managed according to standard practices during the rice growing season without pesticide treatment.

The leaves of the rice plants were collected for quantitative PCR after RBSDV inoculation 7 and 14 days postinoculation (dpi). Total RNA was extracted from the collected leaf samples using TRIzol reagent. The RNA samples were treated with DNase prior to reverse transcription. Quantitative PCR was performed using SYBR Premix Mix (Takara). The relative expression of RBSDV S10 was calculated using the 2^−ΔΔCT^ method (Livak and Schmittgen, 2001). The expression of the rice 18S rRNA gene was used as an internal control for RT‒qPCR. The primers used for RT‒qPCR are listed in Table 1. Three biological replicates evaluated used for each treatment.

At 30 days after the plants were transplanted, symptoms of rice black-streaked dwarf disease were observed. Individuals with the typical symptoms of rice black-streaked dwarf disease were considered susceptible, while those without symptoms of rice black-streaked dwarf disease were considered resistant. DI was evaluated using the following formula: number of RBSDV-infected plants/total number of plants counted × 100 (Wang et al., 2021). Two surveys of incidence were conducted after transplantation—at 30 and at 50 days.

## Data Availability

The raw data supporting the conclusion of this article will be made available by the authors, without undue reservation.
